# Child developmental delay and its associated factors among children aged 12–59 months in Dembecha district, Northwest Ethiopia: a community-based cross-sectional study

**DOI:** 10.3389/fpubh.2024.1464121

**Published:** 2024-12-20

**Authors:** Adugna Kerebh, Melese Linger Endalifer, Molla Yigzaw Birhanu, Animut Takele Telayneh, Lake Kumlachew Abate, Zemene Adissie, Ayenew Negesse, Alehegn Aderaw Alamneh

**Affiliations:** ^1^Dega Damot Health Office, Feresbet, Ethiopia; ^2^Department of Human Nutrition, College of Medicine and Health Sciences, Debre Markos University, Debre Markos, Ethiopia; ^3^Department of Public Health, College of Medicine and Health Sciences, Debre Markos University, Debre Markos, Ethiopia; ^4^Department of Environmental Health, College of Medicine and Health Sciences, Debre Markos University, Debre Markos, Ethiopia

**Keywords:** prevalence, associated factors, children, developmental delay, Dembecha, Ethiopia

## Abstract

**Background:**

Developmental delay is a group of disorders that cause common deficits of adaptive and intellectual function in children. It happens when a child fails to achieve one aspect of developmental skills. Limited information is available regarding the prevalence of developmental delay among children aged 12–59 months in the study area. Therefore, this study aimed to assess the prevalence of developmental delay and its associated factors among this population.

**Methods:**

A community-based cross-sectional study was conducted in Dembecha district among 702 children aged 12–59 months. Data were gathered through face-to-face interviews, and by taking anthropometric measurements using a pretested structured questionnaire. Data were entered into Epi Data version 4.2 software and exported into Statistical Package for Social Science (SPSS) version 25 software for analysis. The WHO Anthro software was used to analyze anthropometric-related data. Bivariable and multivariable binary logistic regression analyses were done to identify factors associated with developmental delay. The odds ratio with a 95% Confidence Interval (CI) was estimated to determine the strength of the association.

**Results:**

The prevalence of developmental delay among children was 26.7% (95% CI: 23.5, 30.2). Toddler child age (AOR = 2.60; 95% CI: 1.42, 4.87), low birth weight (LBW; AOR =4.90; 95% CI: 2.14, 11.48), cesarean section mode of delivery (AOR = 8.60; 95% CI: 3.93, 18.65), preterm delivery (AOR = 2.5; 95% CI: 1.28, 4.74), early initiation of complementary feeding (AOR = 8.40; 95% CI: 3.61, 19.63), stunting (AOR = 2.90; 95% CI: 1.67, 5.22) inadequate meal frequency (AOR = 3.20; 95% CI: 1.74, 5.94), and inadequate dietary diversity (AOR = 3.10; 95% CI: 1.68, 5.85) were significantly associated with child developmental delay.

**Conclusion:**

The prevalence of developmental delay among children was high in Dembecha district compared to the global prevalence. Child developmental delay was associated with toddler child age, LBW, cesarean section mode of delivery, preterm delivery, initiation of complementary feeding before 6 months, stunting, inadequate meal frequency, and inadequate dietary diversity. Therefore, preventing preterm delivery and LBW, initiating complementary feeding before 6 months, stunting, and achieving the minimum meal frequency, and minimum dietary diversity are recommended to prevent child developmental delay.

## Introduction

Child development refers advancement of the child in all areas of human functioning: social and emotional, cognitive, communication and movement (1). The first 1,000 days (from conception to 2 years) is a critical period for brain development (2). The majority of organ development, including the nervous system occurs before 5 years of age. About 90% of the size of the child’s brain grows in the first 5 years of a child’s life (3). Thus, early childhood is the most vulnerable period for the occurrence of developmental delays (4). Delayed child development refers to a child who does not achieve the developmental skills expected of his or her age compared to other children of the same age (5, 6). The American Academy of Pediatrics recommends screening children for developmental delay at 9, 18, and 30 months of age with standardized tools (7). The standardized tools that are widely used to screen children for developmental delay in low-income countries, including Ethiopia are the Denver Developmental Screening Test II (8), the third edition of Ages, and Stages Questionnaires (ASQ-3) (9), and the Bayley Scale of Infant Development III (10).

Delayed child development is a global, regional, and national problem. Globally, 8.4% of children younger than 5 years had developmental disabilities in 2016. Almost all (95%) of these children lived in low-income and middle-income countries (11). According to global statistics, about 5–16% of children around the world have developmental disorders. Approximately 30–50% of these disorders are not identified until school age and therefore cannot be treated (12). More than 250 million, approximately 43% of children living in low-income and middle-income countries have some form of developmental delay, where the burden is highest in Sub-Saharan Africa accounting for 66% in 2010 (13–15).

Developmental delays affect a child’s learning ability and emotional development which negatively impact income or productivity, and their future life. Poor growth and undernutrition are also associated with delayed mental development which leads to poor school performance and reduced intellectual achievement (16). Globally, the average loss of income or productivity due to poor child development is estimated at 19.8% (15).

Many factors, including poor health of pregnant mothers, birth complications, very low birth weight, infections, genetic characteristics, exposure to toxins, trauma, and perhaps low socioeconomic status (17, 18), an increase in the use of electronic devices, even among young children (19) and internal migration (20) are related to increased risk of developmental delay.

In Ethiopia, the burden of child developmental delay cannot be explained solely by poor health or undernutrition of young children, further aggravated by the lack of sensitive and responsive child care, feeding, stimulation, and safety or security, all of which result in an estimated 59% of children under 5 years of age being at the risk of suboptimal development in the country (21).

In Ethiopia, the Early Childhood Development (ECD) initiative strategic plan is being implemented under the child health program aiming that all children grow and thrive in a secure, safe, and nurturing environment (22). In addition, system and policy changes can influence access to health care for the prevention and treatment of illness, malnutrition, early marriage, and maternal education, all of which may affect child development (23).

Though the above strategies are being implemented, the prevalence of developmental delay among children in Ethiopia remains a public health problem. Therefore we aimed to assess the prevalence of developmental delay and its associated factors among children aged 6–59 months in Dembecha district, Northwest Ethiopia.

## Materials and methods

### Study area

The study was conducted in Dembecha district which is one of the 15 districts in the West Gojjam Zone, Amhara Regional State in Northwest Ethiopia. It is located 350 km from Addis Ababa in the Northwest direction, 203 km from Bahir Dar in the South direction, and 38 km from Finote Selam in the East direction. There were 6 health centers and 27 health posts (the lowest administrative primary health care unit that provides basic health services to the community in Ethiopia), which provide basic health services to the people of the district by focusing on maternal and child health. The climatic condition was 83% woyna dega (temperate zone with an altitude of 1,500-2500 m above sea level) 11% dega (vegetated cool zone with an altitude of over 2,600 m above sea level) and 6% Qola (hot zone with an altitude of below 1,500 m above sea level). The population of Dembecha district mostly generates income through agriculture. The total population of the Dembecha district was 175,520 in 2022. There were 40,818 households in the district. In 2022, the estimated number of children between 12 and 59 months old in the district was 18,309 (24).

### Study design, period, and population

A community-based cross-sectional study design was employed from April 19 to May 25, 2022. The study population was all randomly selected children aged 12–59 months in Dembecha district, during the study period. All children aged 12–59 months in the randomly selected kebeles (the smallest administrative unit in Ethiopia) during data the collection period were included in the study. Children with visual or hearing impairment were excluded from the study since they could not perform the specific developmental domains during the assessment.

### Sample size determination and sampling procedures

The sample size was calculated using a single population proportion formula by considering 29.4% of the developmental delay taken from a previous study in Gurage Zone, Southwest Ethiopia (25) and 95% confidence interval, 5% marginal error, a design effect of 2, and 10% non-response rate which resulted in 702.

A multistage sampling technique was used to select the study participants. First, one urban kebele and seven rural kebeles were selected using a simple random sampling method. Then, the samples were distributed proportionally based on size. Finally, participants in each kebeles were selected using a systematic sampling technique after calculating the sampling interval for each kebeles The sample frame was the list of households with children 12–59 months of age. A list of households with children was obtained from a family folder, found in the health post. In households with two children less than 5 years of age, one was selected randomly by lottery. A revisit of three times was made in a case where eligible respondents were not available by the time of data collection and those who were not available after the revisit were considered as non-respondent.

### Study variables

The dependent variable was the child’s developmental status categorized as normal or delayed.

The independent variables were:

- Socio-demographic characteristics (maternal age, residence, religion, marital status, maternal educational status, maternal occupation, father’s occupation, father’s educational status, household income level, and family size),
- Child-related factors (child age, child sex, birth order, birth weight, complementary feeding, minimum meal frequency, minimum dietary diversity, wasting, stunting, underweight, and history of comorbidities),
- Maternal-related factors (medication during the pregnancy, diseases during pregnancy, alcohol drinking during pregnancy, and contraceptive utilization), and.
- Obstetric health care and health-service related factors (place of delivery, parity, multiple pregnancies, gestational age, mode of delivery, and hypertension disorder during pregnancy).

### Operational definitions

**Developmental delay**: It was defined as a child whose ASQ-3 score fell below the cut-off points for their age in any ASQ-3 domains. The five critical domains in a child’s development are communication, gross motor, fine motor, problem-solving, and personal social (9, 26) (Table 1).

**Table 1:** Cut-off points of each child development milestone for different age categories to classify a child as high risk to developmental delay, needs monitoring, and on schedule based on ASQ-3 scores.

Age in month	Area	High risk	Need monitoring	On schedule
12 months	Communication	< 16	16–30	>30
	Gross motor	< 21	21–35	>35
	Fine motor	< 34	34–42	>42
	Problem solving	< 27	27–38	>38
	Personal social	< 22	22–34	>34
13 months 0 day–14 months 30 days	Communication	< 17	17–31	>31
	Gross motor	< 26	26–39	>39
	Fine motor	< 23	23–35	>35
	Problem solving	< 23	23–34	>34
	Personal social	< 23	23–35	>35
15 months 0 day–16 months 30 days	Communication	< 17	17–30	>30
	Gross motor	< 38	38–47	>47
	Fine motor	< 32	32–42	>42
	Problem solving	< 31	31–41	>41
	Personal social	< 26	26–37	>37
17 months 0 day–18 months 30 days	Communication	< 13	13–30	>30
	Gross motor	< 37	37–41	>41
	Fine motor	< 34	34–43	>43
	Problem solving	< 26	26–36	>36
	Personal social	< 27	27–32	>32
19 months 0 day–20 months 30 days	Communication	< 21	21–34	>34
	Gross motor	< 40	40–47	>47
	Fine motor	< 36	36–44	>44
	Problem solving	<29	29–38	>38
	Personal social	< 33	33–42	>42
21 months 0 day–22 months 30 days	Communication	< 13	13–30	>30
	Gross motor	< 28	28–39	>39
	Fine motor	< 30	30–39	>39
	Problem solving	< 29	29–39	>39
	Personal social	< 30	30–40	>40
23 months 0 days–25 months 15 days	Communication	< 25	25–37	>37
	Gross motor	< 38	38–46	>46
	Fine motor	< 35	35–44	>44
	Problem solving	< 30	30–44	>44
	Personal social	< 32	32–41	>41
25 months 16 days–28 months 15 days	Communication	< 24	24–37	>37
	Gross motor	< 28	28–39	>39
	Fine motor	< 18	18–31	>31
	Problem solving	< 28	28–38	>38
	Personal social	≤25	26–36	>36
28 months 16 days–31 months 15 days	Communication	≤ 33	34–43	>43
	Gross motor	≤36	37–46	>46
	Fine motor	≤19	20–33	>33
	Problem solving	≤27	28–37	>37
	Personal social	≤32	33–42	>42
31 months 16 days–34 months 15 days	Communication	≤25	26–36	>36
	Gross motor	< 35	35–44	>44
	Fine motor	< 12	12–23	>23
	Problem solving	< 27	28–43	>43
	Personal social	≤29	30–40	>40
34 months 16 days–38 months 30 days	Communication	≤31	32–42	>42
	Gross motor	≤37	38–46	>46
	Fine motor	≤18	19–32	>32
	Problem solving	≤30	31–41	>41
	Personal social	≤35	36–44	>44
39 months 0 day–44 months 30 days	Communication	< 27	27–38	>38
	Gross motor	≤36	37–45	>45
	Fine motor	< 20	20–34	>34
	Problem solving	≤28	29–40	>40
	Personal social	≤ 31	32–42	>42
45 months 0 day–50 months 30	Communication	< 31	31–42	>42
	Gross motor	≤32	33–43	>43
	Fine motor	< 16	16–31	>31
	Problem solving	≤31	32–42	>42
	Personal social	< 27	27–37	>37
51 months 0 day–56 months 30 days	Communication	< 32	32–43	>43
	Gross motor	≤35	36–44	>44
	Fine motor	< 17	17–32	>32
	Problem solving	≤28	29–39	>39
	Personal social	≤32	33–43	>43
57 months 0 day−59 months 0 day	Communication	≤33	34–43	>43
	Gross motor	< 31	31–41	>41
	Fine motor	< 27	27–39	>39
	Problem solving	< 30	30–41	>41
	Personal social	< 39	39–47	>47

**Stunting**: Children with a height-for-age at Z-score below −2 SD of the median value of the World Health Organization (WHO) international standard reference (27).

**Wasting**: Children with a weight-for-height Z-score below −2 SD of the median value of the WHO international standard reference (27).

**Underweight**: Children with weight-for-age Z-score below −2 SD of the median value of the WHO international standard reference (27).

**Minimum dietary diversity**: The percentage of children 12–59 months who received foods from 4 or more food groups during the previous day. The seven food groups used for tabulation of this indicator are grains, roots, and tubers; legumes and nuts; dairy products (milk, yogurt, and cheese); flesh foods (meat, fish, poultry, and liver/organ meats); eggs; vitamin A-rich fruits and vegetables; and other fruits and vegetables (28).

**Complementary feeding**: The process of introducing solid, semi-solid, or soft foods in addition to breast milk at 6 months of age (28).

**Minimum meal frequency**: Percentage of children 12–59 months of age who consumed solid, semi-solid, or soft foods at least the minimum number of times during the previous day. The minimum number of times was 4 times of meals per day for 12–59 months of children (28).

### Data collection tool and procedure

A structured paper-based questionnaire was used to collect data on socio-demographic, maternal-related factors, child health-related factors, nutritional variables, and food access at the household level. It was constructed by adapting and modifying the Ethiopia Mini Demographic and Health Survey (29) 2019 and previous research on a similar topic (25, 30). First, the English version questionnaire was prepared. Then it was translated to the native language Amharic and was back translated to the English language.

The child development assessment was done using the ASQ-3. The ASQ-3 has five subscales: communication, gross motor, fine motor, problem-solving, and personal-social. The ASQ-3 was answered with 10 points for “yes,” 5 points for “sometimes,” and 0 points for “not at all.” Each child was asked 30 questions and assessed out of 300 scores; and six questions out of 60 scores for each development milestone. Each domain was classified into three (high risk for developmental delay, needs monitoring, and on schedule) for each age category based on ASQ-3. Finally, child development was categorized as developmental delay and normal development (needs monitoring and on schedule). Developmental delay refers to a high risk of developmental delay whereas normal development refers to a low-to-moderate risk (9, 25, 26) (Table 1).

The ASQ-3 had a validity of 0.83–0.88, reliability of 0.90–0.94, sensitivity of 0.38–0.91, and specificity of 0.39–0.95 (26, 31). This tool was used in a study conducted in the Wolaita Sodo and Gurage zones in South Ethiopia. Also, the ASQ-3 developmental assessment tool was used by 56.3% of health professionals who were working at public hospitals in Addis Ababa, Ethiopia, 2018 (32).

Children’s dietary diversity was assessed with 7 food groups by using the WHO infant and young child feeding indicator assessment tool with some modifications to fit the context based on the last 24-h recall method (28).

Data was collected from caregivers or mothers of the children by two public health professionals and three health extension workers who could communicate well in the local language. The face-to-face interviews were used to collect data for socio-demographic factors and child-related factors. However, most parts of the developmental questions were assessed when a child performed or failed the activity required. The supervisors had oversight over the data collection process and the data collectors and supervisors had regular meetings with the principal investigator at the end of each day of the data collection period.

Anthropometric measurements for height/length were collected as per the World Health Organization (WHO) guidelines. A portable stadiometer was used to measure the height of older children (above 2 years) and a calibrated length board was used for younger children (below 2 years). Older children were measured in a standing position, while younger children below 2 years old were measured in a recumbent position. Children’s height and weight were measured without shoes, hats, and hair ornaments. When measuring height, a child’s head, shoulders, buttocks, and heels were attached to the vertical surface of the stadiometer. The height measurement was recorded to the nearest 0.1 cm. The weight of each child was measured while barefoot and light clothing and recorded to the nearest 0.1 kg using weighing scales (33). All variables apart from anthropometric measurements and observed ASQ-3 measures were self-reported by the mother/caregiver of a child.

### Data quality assurance

The quality of the data was maintained by translating the English version questionnaire into the Amharic language, then it was translated back to English to check its consistency. To assess the appropriateness of wording, clarity of the questions, and respondent reaction to the questions, a pre-test was conducted on 5% of the calculated sample size in the Dega Damot district which had similar basic socio-economic characteristics, and the necessary amendments were made. Before data collection, data collectors and supervisors were trained for 1 day by the principal investigator about the overall purpose of the study, how to approach the study participants, and the way to collect data. During the data collection time, close supervision and monitoring were carried out by supervisors and the principal investigator to ensure the quality of the data. Finally, the supervisor and investigator checked the data for accuracy, clarity, consistency, and completeness daily.

### Data processing and analysis

The collected data was checked for completeness. The pre-coded data was entered into Epi data version 3.1 statistical software. Then the data was exported to SPSS version 25 for analysis. Descriptive analysis such as frequency, percentage, mean and standard deviation was computed, and the results were presented using texts, crosstabs, tables, and figures. The child’s developmental status was computed for each milestone as delayed and normal development and the five milestones were combined to determine the child’s overall developmental status. Finally, child development was categorized as developmental delay and normal development. WHO Anthro version 1.0.4 software was used to convert the anthropometric measure weight, height, and age value into Z-score of the indices HAZ, WAZ, and WHZ. Children whose height-for-age, weight-for-height, and weight-for-age < −2 SD from the median of the reference population were considered stunted, wasted, and underweight, respectively. Binary logistic regression was performed to identify factors significantly associated with child development delay. Those variables with a *p*-value <0.25 in bivariable analysis were entered into the multivariable analysis to control for possible confounding variables and to examine the association. The strength of association was measured using the adjusted odds ratio with 95% confidence intervals. Statistical significance was declared at a *p*-value of <0.05. A good fitness of the model was observed from the Hosmer Lemeshow test. The absence of multicollinearity was checked by using variance inflation factors and tolerance. The variables variance inflation factor and tolerance were < 10 and > 0.1, respectively.

### Ethical consideration

An ethical clearance letter was obtained from the Research Ethics Committee of the College of Health Science of Debre Markos University (Reference Number: HSC/R/C/Ser/PG/Co/118/11/14). A written official letter along with the ethical clearance was submitted to Dembecha District Health Office by the principal investigator to get permission from the Health office before the data collection. In addition, written informed consent was taken from the mothers of the children. While conducting the study, children who were found to be malnourished, their mothers were informed and advised about the measures to be taken, and were linked to the nearby public health services for treatment and support. The obtained information was kept confidential by locking it with a password.

## Results

This study approached 702 mothers with children aged 12–59 months in Dembecha district, with a 98.00% response rate (688 mothers with children). The average age of the respondents was 31.28 years, and 274 (39.80%) were between the ages of 25 and 34. The majority of respondents were from rural areas (73.7%), Ethiopian Orthodox Tewahedo church followers (93.6%), married (85.6%), and had a family size of less than five (52.6%). About 85.2% of mothers were housewives, while 74.3 and 34.9% of fathers were farmers and had a primary education, respectively (Table 2).

**Table 2:** Socio-demographic characteristics of the mothers in Dembecha district, Northwest Ethiopia, 2022 (*N* = 688).

Variable	Characteristics	Frequency (%)
Age of the mother	Young(15–24)	162 (23.5)
	Middle(25–34)	274 (39.8)
	Late(≥35)	252 (36.6)
Residence	Rural	507 (73.7)
	Urban	181 (26.3)
Religion	Orthodox Tewahdo	644 (93.6)
	Muslim	44 (6.4)
Marital status	Married	589 (85.6)
	Divorced	27 (3.9)
	Widowed	32 (4.7)
	Separated	40 (5.8)
Maternal education status	Cannot read and write	114 (16.6)
	Read and write	221 (32.1)
	Primary education	266 (38.7)
	Secondary education	67 (9.7)
	More than secondary education	20 (2.9)
Occupation of the mother	Housewife	586 (85.2)
	Merchant	77 (11.2)
	Daily laborer	3 (0.4)
	Government employee	22 (3.2)
Education level of the father	Cannot read and write	68 (10.5)
	Read and write	141 (21.9)
	Primary education	225 (34.9)
	Secondary education	172 (26.7)
	More than secondary education	39 (6.1)
Occupation of the father	Farmer	479 (74.3)
	Merchant	86 (13.3)
	Daily laborer	32 (5.0)
	Government employee	29 (4.5)
	Self-employed	19 (3.0)
Average household monthly income	Less than mean(<935 ETB)	476 (69.2)
	Above mean(≥935 ETB)	212 (30.8)
Family size	<5	362 (52.6)
	≥5	326 (47.4)

### Maternal obstetric and healthcare-related characteristics

About 96.51% of children were born in a health facility. The majority of children (65.0%) were born at term. More than two-thirds (69.9%) of the children were born via spontaneous vaginal delivery. Most of the mother (95.4%) did not develop hypertension during pregnancy. Half of the mothers (51.3%) had no history of illness or disease during pregnancy. More than half of the mothers (57.9%) drank alcohol while pregnant (Table 3).

**Table 3:** Maternal and obstetric health care with health service utilization related factors in Dembecha district (*N* = 688), 2022.

Variable	Characteristics	Frequency (%)
Medication during pregnancy	Yes	331 (48.1)
	No	357 (51.9)
Diseases during pregnancy	Yes	335 (48.7)
	No	353 (51.3)
Alcohol drinking during pregnancy	Yes	398 (57.9)
	No	290 (42.2)
Contraceptive use	Yes	147 (21.4)
	No	541 (78.6)
Place of delivery	Health institution	664 (96.5)
	Home	24 (3.5)
Mode of delivery	Spontaneous vaginal delivery	481 (69.9)
	Instrumental delivery	119 (17.3)
	Caesarean section (CS)	88 (12.8)
Multiple pregnancy	Yes	14 (2.0)
	No	674 (98.0)
Gestational age	Below 9 months	125 (18.2)
	At 9 months	447 (65.0)
	Above 9 months	116 (16.9)
Parity	<5	637 (92.6)
	≥5	51 (7.4)
Hypertension disorder pregnancy	Yes	32 (4.7)
	No	656 (95.4)

### Socio-demographic, feeding practice and nutritional status of children

Three hundred eighty-four (55.8%) children were females. The mean age of the children was 31.0 months (SD = 12.4 months). Two-thirds (66.6%) of the children were toddlers aged 1–3 years. More than three-fourths (87.8%) of children were born with normal birth weight. In terms of feeding, 373 (54.2%) began complementary feeding at the age of 6 months. Less than half of children (41.0%) eat less than four times per day. In the previous 24 h, 57.0% of children met the minimum dietary diversity (four food groups; Table 4).

**Table 4:** Socio-demographic, feeding practice of children in Dembecha district (*N* = 688) 2022.

Variable	Characteristics	Frequency (%)
Age of the child	Toddler (1–3 years)	458 (66.6)
	Preschool (3–5 years)	230 (33.4)
Sex of the child	Female	384 (55.8)
	Male	304 (44.2)
Birth Weight	<2500gram	82 (11.9)
	> = 2,500 gram	604 (87.8)
Infectious diseases within the past 6 months	Yes	228 (33.1)
	No	460 (66.9)
Birth order	First	141 (20.5)
	Second	219 (31.8)
	Third	145 (21.1)
	Fourth and above	183 (26.6)
Initiation of complementary feeding	Below 6 months	90 (13.1)
	At 6 months	373 (54.2)
	Above 6 months	225 (32.7)
Meal frequency (per day)	<4 times	282 (41.0)
	≥4 times	406 (59.0)
Dietary diversity (per day)	<4 food groups	296 (43.0)
	≥4 food groups	392 (57.0)

**Figure 1:**
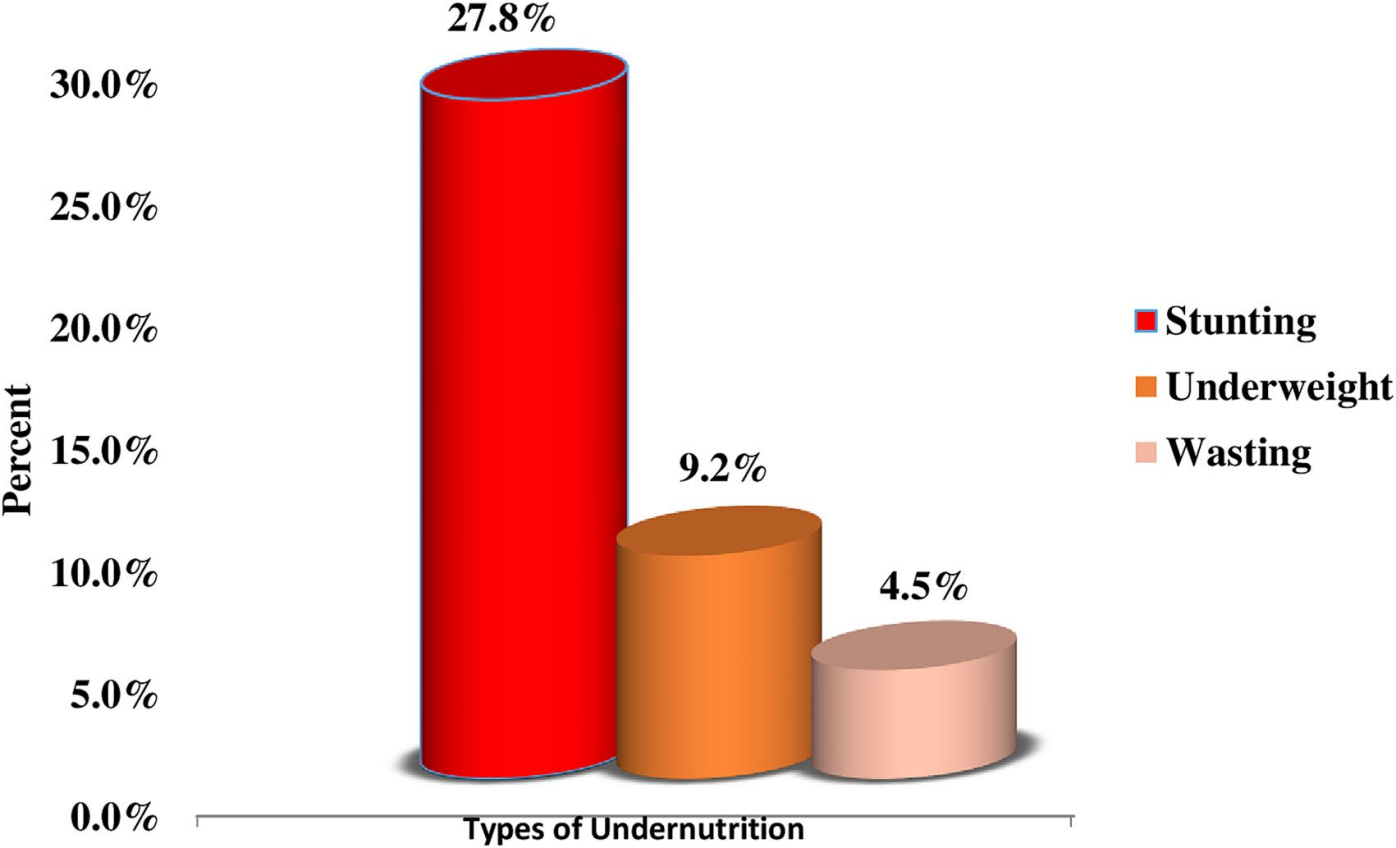
Prevalence of childhood undernutrition among children aged 12–59 months in Dembecha district (*N* = 688), 2022.

Concerning the nutritional status of children, about 27.8% (95% CI: 24.4, 31.3%), 9.2% (95% CI: 7.1, 11.6%), and 4.5% (95% CI: 3.1, 6.3%) were stunted, underweight, and wasted, respectively (Figure 1).

### Prevalence of childhood developmental delay

**Figure 2:**
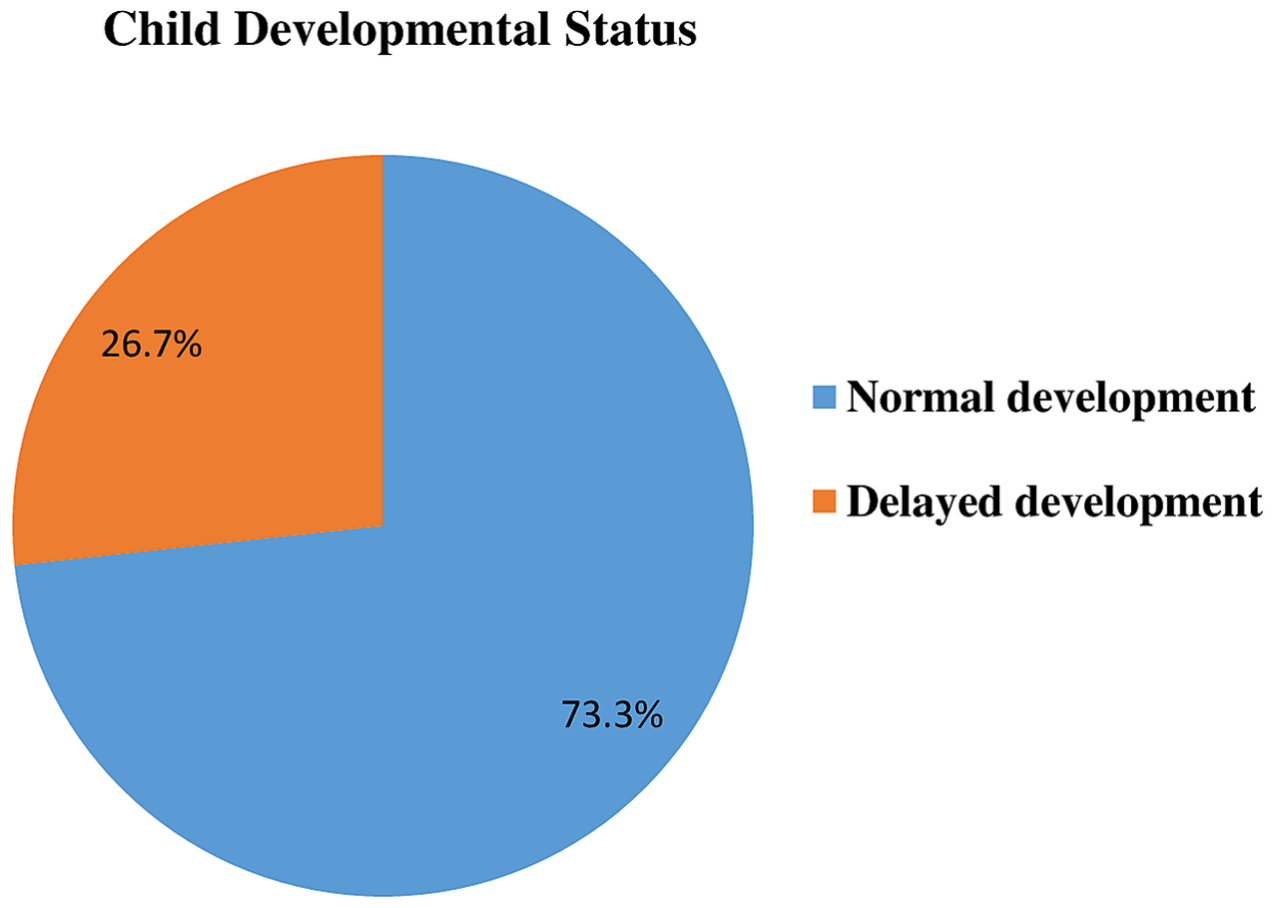
Developmental status in Dembecha district among children’s aged 12–59 months (*N* = 688), 2022.

The mean ASQ-3 score was 155.4, with a standard deviation of 25.7, a minimum score of 80, and a maximum of 270. The mean ASQ-3 score for each domain ranged from 27.2–35.9, with a standard deviation from 5.5–8.2, and a range from 10 to 55 for communication,10 to 60 for fine motor, and 15 to 60 for the remaining of the milestones. This study found that 26.7% (95% CI: 23.5, 30.2%) of children have developmental delays. It is expressed as communication delay (17.2%), gross motor delay (20.6%), fine motor delay (17.4%), problem-solving delay (16.7%), and personal social delay (18.2%). The remaining 73.3% with 95%CI (69.8, 76.5%) were on normal development to their age (Table 5; Figure 2).

**Table 5:** Prevalence of developmental delay in each milestone among children aged 12–59 months in Dembecha district (*N* = 688), 2022.

Domain	Mean ± SD	Prevalence (95% CI)
Communication	28.51 ± 7.2	17.2 (14.4,20.2)
Gross motor	35.86 ± 6.7	20.6 (17.7,23.9)
Fine motor	27.2 ± 8.2	17.4 (14.7,20.5)
Problem solving	31.2 ± 5.5	16.7 (14.0,19.7)
Personal–social	32.65 ± 6.3	18.2 (15.4,21.3)

About 28 (4.1%; 95% CI: 2.7, 5.8%) children had developmental delays in all the five developmental domains.

### Factors associated with childhood developmental delay

Maternal age, marital status, weight at birth, gestational age, time of initiation of complementary feeding, birth order, place of delivery, mode of delivery, child age, stunting, underweight, parity, meal frequency, dietary diversity, and family size were associated with child development delay in bivariable logistic regression analysis and identified as candidates for multivariable logistic regression analysis (Table 6).

**Table 6:** A bivariable and multivariable analysis of factors associated with developmental delay among children aged 12–59 months in Dembecha district, Northwest, Ethiopia (*N* = 688), 2022.

Explanatory variable	Characteristics	Children with developmental delay	COR(95%CI)	AOR(95%CI)
		Frequency (%)		
Maternal age	15–24 years	54 (33.3)	1.15 (0.75,1.76)	4.00 (0.92,17.35)
	25-34 years	54 (19.7)	0.56 (0.38,0.84)	1.40 (0.52,3.59)
	≥35 years	76 (30.2)	1.00	1.00
Marital status	Married	160 (27.2)	1.00	1.00
	Divorced	10 (37.0)	1.57 (0.70,3.51)	1.20 (0.36,4.02)
	Widowed	8 (25.0)	0.89 (0.39,2.03)	0.60 (0.17,2.48)
	Separated	6 (15.0)	0.47 (0.19,1.14)	1.10 (0.23,5.53)
Family size	<5	87 (24.0)	1.00	1.00
	≥5	97 (29.8)	1.33 (0.95,1.87)	0.50 (0.03,6.84)
Child age	12–36 months	141 (30.8)	1.93 (1.31,2.84)	2.60 (1.42,4.87)**
	37–59 months	43 (18.7)	1.00	1.00
Weight at birth	<2500gram	61 (74.4)	11.59 (6.79,19.78)	4.90 (2.14,11.48)***
	≥2500gram	121 (20.0)	1.00	1.00
Birth order	First	40 (28.4)	1.00	1.00
	Second	47 (21.5)	0.69 (0.42,1.12)	0.80 (0.25,1.32)
	Third	32 (22.1)	0.71 (0.41,1.22)	0.70 (0.32,1.70)
	Fourth and above	65 (35.5)	1.39 (0.86,2.23)	1.50 (0.62,3.55)
Place of delivery	Health institution	166 (25.0)	1.00	1.00
	Home	18 (75.0)	9.00 (3.51,23.05)	3.50 (0.92,13.57)
Mode of delivery	Spontaneous vaginal delivery	84 (17.5)	1.00	1.00
	Instrumental delivery	35 (29.4)	1.96 (1.244,3.11)	1.50 (0.78,2.92)
	Caesarean section	65 (73.9)	13.35 (7.85,22.70)	8.60 (3.93,18.65)***
Gestational age	At 9 months	53 (11.9)	1.00	1.00
	Below 9 months	72 (57.6)	10.09 (6.4,15.93)	2.50 (1.28,4.74)**
	Above 9 months	59 (50.9)	7.69 (4.84,12.23)	1.80 (0.93,3.76)
Parity	<5	160 (25.1)	1.00	1.00
	> = 5	24 (47.1)	2.65 (1.48,4.72)	0.60 (0.21,1.57)
Initiation of complementary feeding	At 6 month	17 (4.6)	1.00	1.00
	Below 6 months	54 (60.0)	31.40 (16.49,59.8)	8.40 (3.61,19.63)***
	Above 6 months	113 (50.2)	11.12 (10.16,26.70)	4.60 (0.98,17.42)
Meal frequency	<4 times	139 (49.3)	7.79 (5.29,11.49)	3.20 (1.74,5.94)***
	> = 4 times	45 (11.1)	1.00	1.00
Dietary diversity	> = 4 food groups	39 (9.9)	1.00	1.00
	<4 food groups	145 (49.0)	8.69 (5.81,12.98)	3.10 (1.68,5.85)***
Stunting	Yes	91 (47.6)	3.95 (2.75,5.68)	2.90 (1.67,5.22)***
	No	93 (18.7)	1.00	1.00
Underweight	Yes	33 (52.4)	3.45 (2.03,5.85)	1.90 (0.76,4.55)
	No	151 (24.2)	1.00	1.00

COR, Crude Odd Ratio; AOR, Adjusted Odd Ratio; *p-value < 0.05, **p-value < 0.01, and ***p-value < 0.001.

In multivariable logistic regression analysis; toddler age of the child, LBW, cesarean section mode of delivery, preterm delivery, initiation of complementary feeding before 6 months, stunting, inadequate meal frequency, and inadequate dietary diversity were significantly associated with developmental delay at *p*-value of 0.05 level of significance (Table 6).

Children born with a LBW were nearly 5 times more likely to experience developmental delay than children with a normal birth weight (AOR = 4.90; 95% CI: 2.14, 11.48). Toddlers (1–3 years) were 2.6 more likely to experience developmental delay than children who were in the age of preschool (3–5) years (AOR = 2.60; 95% CI: 1.42, 4.87). Children born by cesarean section were nearly 9 times more likely to have developmental delay (AOR = 8.60; 95% CI: 3.93, 18.65; Table 6).

Children who started complementary feeding before 6 months of age were 8.4 times more likely to have developmental delay than children who started at 6 months (AOR = 8.40; 95% CI: 3.61, 19.63). Preterm children were 2.5 times more likely to experience developmental delay compared to children who were delivered at term (AOR = 2.50; 95% CI: 1.28, 4.74). Children who had meal frequency of <4 times were 3 times more likely to develop developmental delay compared to children who had meal frequency of ≥4 times (AOR = 3.20; 95% CI: 1.74, 5.94; Table 6).

Children who had inadequate dietary diversity were 3 times more likely to develop developmental delay compared to children who received more than four food groups (AOR = 3.10; 95% CI: 1.68, 5.85). The odds of developmental delay among stunted children were nearly 3 times higher as compared to normal children (AOR = 2.90; 95% CI: 1.67, 5.22; Table 6).

## Discussion

This study aimed to assess child developmental delay and its associated factors. This study found that the prevalence of child developmental delay was 26.7%. Developmental delay among children was associated with toddler child age, LBW, cesarean section mode of delivery, preterm delivery, initiation of complementary feeding before 6 months, stunting, inadequate meal frequency, and inadequate dietary diversity.

The prevalence of child developmental delay in this study was in line with studies conducted in Ethiopia (29.4%) (25) and Iran (26.3%) (34).

However, the prevalence of child developmental delay in this study was higher compared to other studies conducted in Ethiopia (19%) (35), Central Iran (11.8%) (12), and the global prevalence (8.4%) (11). The discrepancy might be due to the better socio-economic status, and the child health care system in Iran. The difference from the previous Ethiopian study might be attributed to the high prevalence of malnutrition in the current study area (30), which results in developmental delay (35). On the contrary, the prevalence of child developmental delay in this study was lower compared to studies conducted in Nepal (56.4%) (5), South Africa (55.2%) (36), and Nigeria (35.4%) (37). The possible reasons for this variation might be attributed to several factors including the study method used, the different socio-economic classes of participants, differences in study settings, and different tools for assessing developmental milestones. In addition, the high prevalence of child developmental delay in Nepal might be due to the inclusion of urban slum areas in their study. Children living in slum areas have a higher risk of undernutrition (38, 39), and Human Immuno-deficiency virus (HIV) infection (40), ultimately contributing to child developmental delay.

Moreover, the prevalence of developmental delay was lower compared to the national estimate of Ethiopia (59%) (21) and the Lancet 2016 report from the world which was 43% of children under 5 years of age living in low- and middle-income countries being at risk of suboptimal development (4). The difference from the Lancet Report is attributed to the inclusion of a wide population of the globe. The current study shows children aged 1–3 years had higher odds of developmental delay as compared to 3-5-years-old children. This finding is supported by a study in Pakistan (41). The probable reason might be infants and toddlers grow and develop rapidly in the first 2 years, making them particularly vulnerable or influenced by nutritional inadequacies, and environmental and socio-demographic variables. A vital window of opportunity for ensuring children’s proper growth and development through adequate nutrition occurs throughout the first 2 years of life. It can also be due to the cultural aspect that young children are mostly kept indoors and therefore have less exposure and stimulation.

In this study, children with LBW have a higher likelihood of developing developmental delay compared to children with normal birth weight which is similar to a study done in West Bengal (42). Because they are more likely to have neuro-developmental abnormalities, children with lower birth weights are likely to have a higher chance of experiencing developmental delays. It is reported that approximately 10% of the extremely LBW (< 1,000 grams) children will develop cerebral palsy which is a disorder of movement, posture, and balance (43). Also, a 32% rate of cerebral palsy is found in those infants weighing less than 1,500 grams (44). Low birth weight remains to be a risk factor for developmental delay which can easily be prevented by regular monitoring and better nutrition.

In this study, the cesarean section mode of delivery had higher odds of developmental delay in children which was similar to other studies done in Brazil (45). The possible reason for this increment might be associated with the absence of contact with the mother’s natural bacterial flora during a cesarean section delivery. Vaginally delivered babies are exposed to microbes residing in the maternal birth canal. Microbial colonization of the infant gastrointestinal tract is important for the development of gut immunology, brain-gut-axis, and nervous system, influencing brain function, and behavior (46, 47). In addition, neonates delivered through cesarean section have a high risk of birth asphyxia (48, 49), which contributes to child developmental delay (50).

Premature birth in the current study was associated with a higher risk of developmental delay compared to term birth, which is similar to other studies conducted in Turkey (51), North India (52), and Southwest Ethiopia (25). The probable explanation for the increase of child developmental delay in preterm children is due to an increased risk of hypoxic–ischemic events that often lead to severe cognitive developmental delays, or motor impairments (53). Preterm children are also at risk of intraventricular bleeds and infections likely to result in neural injury as well (54).

This study also showed that children who started complementary feeding before 6 months had increased odds of developmental delay. The evidence was similar to the findings from Saudi Arabia (35, 55), and Ethiopia (25). Babies who start complementary feeding early have higher rates of iron deficiency anemia because it can decrease iron absorption from breast milk. Iron is an important element for brain growth and development as demonstrated in the fetuses and neonates (56). In addition, feeding other than breast milk causes early satiety which interferes with the infant’s feeding behavior, decreases the frequency of breastfeeding, or reduces breast milk production. Since human milk contains fatty acids like arachidonic acid and docosahexaenoic acid, it is important for the appropriate development of a baby’s organs, tissues, and brain development. The essential fatty acids enhance the production and activity of various immune cells, including lymphocytes and phagocytes (57, 58). Thus, exclusive breastfeeding for up to 6 months decreases the risk of infections in infants compared to early initiation of complementary feeding for infants (59).

The current study showed that stunted children had higher odds of developmental delay. This finding is consistent with the study conducted in Ethiopia (25, 35), Nigeria (37), and Nepal (5). This is because malnutrition results in tissue damage, growth retardation, disorderly differentiation, reduction in synapses and synaptic neurotransmitters, delayed myelination, and reduced overall development of the brain. Inadequate brain growth explains why malnourished children suffer from behavioral and cognitive deficits, including slower language and fine motor development. Because they are lethargic and apathetic, malnourished children have a problem understanding the information and they are less interested in their surroundings than well-nourished children. This results in delayed social interaction skills (60). Children who are malnourished also have deficiencies in micronutrients, like calcium and vitamin D, which are critical for the health of their skeletal muscles. Therefore, a lack of certain micronutrients may have an impact on motor abilities. Nutritional insufficiencies at an acute stage may damage the cognitive profile and entire auditory system in children, resulting in verbal and written language problems (37).

Children with inadequate meal frequency (<4 times a day) have increased odds of child developmental delay. This evidence was supported by findings from Ethiopia (35) and China (61). In order to promote brain growth, which is marked by intense myelination throughout the first 5 years of life, children need a lot of energy from food. Failure to meet energy needs may lead to changes in metabolism in the brain and decreases in muscle strength, thus affecting the child’s ability to develop normal gross motor skills (61).

Furthermore, the current study also shows that low dietary diversification had higher odds of child developmental delay. This result is comparable with the study findings in (35) Ethiopia (25), and China (61). The relationship between dietary diversity and child developmental outcomes might be explained by several mechanisms, including increased intake of micronutrients and protein, greater amounts and variety of psychosocial stimulation, and building muscles for enhanced motor skills, physical activity, and social function, which may, in turn, enrich the child’s interaction with and exploration of the environment, ultimately contributing to the development of problem-solving skills. The increased risk for developmental delay due to low diversification is that low dietary diversity causes nutrient deficiency like protein, energy, fats, iron, zinc, copper, iodine, selenium, vitamin A, choline, and folate (62). Therefore, brain development, both structural and functional, is highly dependent on an adequate supply of protein and micronutrients.

The findings should be interpreted considering the following limitations; Recall bias might be introduced during the collection of child birth weight, age at initiation of complementary feeding, and gestational age since it depends on the respondents’ memory. In addition, we did not collect data on the breastfeeding versus formula-feeding status of a child, maternal height, and maternal mental health status. As a result, the study findings have not been adjusted for these variables. Thus, future researchers shall consider these factors in their study. Furthermore, because the study was cross-sectional, it was unable to demonstrate a cause-and-effect relationship. Moreover, there have not been studies exploring the reliability and validity of the ASQ-3 for Ethiopia, as a result, the results should be interpreted with this limitation in mind.

## Conclusion

In conclusion, the prevalence of child developmental delay was high in Dembecha district as compared to the global prevalence. Child development delay was associated with toddler child age, LBW, cesarean section mode of delivery, preterm delivery, initiating complementary feeding before 6 months, stunting, inadequate meal frequency, and inadequate dietary diversity. Therefore, preventing preterm delivery, initiation of complementary feeding before 6 months, stunting and achieving the minimum meal frequency, and minimum dietary diversity are recommended to prevent developmental delay among children aged 12–59 months.

## Data Availability

The raw data supporting the conclusions of this article will be made available by the authors, without undue reservation.
